# HIF-1α- Targeting Acriflavine Provides Long Term Survival and Radiological Tumor Response in Brain Cancer Therapy

**DOI:** 10.1038/s41598-017-14990-w

**Published:** 2017-11-02

**Authors:** Antonella Mangraviti, Tula Raghavan, Francesco Volpin, Nicolas Skuli, David Gullotti, Jinyuan Zhou, Laura Asnaghi, Eric Sankey, Ann Liu, Yuan Wang, Dong-Hoon Lee, Noah Gorelick, Riccardo Serra, Michael Peters, Destiny Schriefer, Fabien Delaspre, Fausto J. Rodriguez, Charles G. Eberhart, Henry Brem, Alessandro Olivi, Betty Tyler

**Affiliations:** 1 Department of Neurosurgery, Johns Hopkins University, Baltimore, MD USA; 2 Department of Radiology, Johns Hopkins School of Medicine, Baltimore, MD USA; 3 Department of Pathology, Johns Hopkins School of Medicine, Baltimore, MD USA; 4 McKusick-Nathans Institute for Genetic Medicine, Johns Hopkins University, Baltimore, MD USA; 5 Department of Oncology, Johns Hopkins University, Baltimore, MD USA; 6 Department of Ophthalmology, Johns Hopkins University, Baltimore, MD USA; 7 Department of Biomedical Engineering, Johns Hopkins University, Baltimore, MD USA; 8 Department of Neurosurgery, Catholic University School of Medicine, Rome, Italy; 9 Faculty of Health Sciences and Brain & Mind Centre, The University of Sydney, Sydney, NSW Australia

## Abstract

Tumor progression, limited efficacy of current standard treatments, and the rise in patient mortality are associated with gene expression caused by the synergistic action of intratumoral hypoxia and HIF-1α activation. For this reason, recent investigations have focused on HIF-targeting therapeutic agents, with encouraging preclinical and clinical results in solid tumors. Here we describe the efficacy of a HIF-1α inhibitor, Acriflavine, and demonstrate its potency against brain cancer. This safe antibacterial dye induces cell death and apoptosis in several glioma cell lines, targets HIF-1α–mediated pathways, and decreases the level of PGK1, VEGF and HIF-1α *in vitro* and *in vivo*. Administered locally via biodegradable polymers, Acriflavine provides significant benefits in survival resulting in nearly 100% long term survival, confirmed by MRI and histological analyses. This study reports preclinical evidence that this safe, small molecule can contribute to brain tumor therapy and highlights the significance of HIF-1α-targeting molecules.

## Introduction

Primary malignant central nervous system (CNS) tumors are the leading cause of cancer death in children and adolescents (aged birth to 19 years) and the third in young adults (aged 15 to 39), with nearly 17,000 people estimated to lose their life with these tumors in 2017^1,2^. Glioblastoma (GBM), a heterogeneous and highly invasive diffuse grade IV^3^ glioma, recently classified by three different variants based on IDH and H3K27M mutations, despite aggressive multimodal therapeutic approaches, has the worst survival at 5 years^2^ (5.1%) and a worsening annual incidence rate^4,5^. Recent studies on the mechanism of brain tumor growth have found a clear association between the overexpression of hypoxia-induced genes and increased probability of invasion, high recurrence, and poor clinical outcomes^6,7^. These complex and adaptive signaling hypoxia-mediated pathways make brain tumor cells uniquely resistant to chemotherapy^8^ due to enhanced migrating capacity^9^ and resistance to apoptosis^10^. These pathways require the transcriptional activity of hypoxia-inducible factor 1 (HIF-1) which acts as a “crucial switch” promoting glycolysis and neoangiogenesis^11^, oncogene signaling, and a host of metabolic, survival, and proliferative effects necessary for brain tumor growth^12,13^. As such, HIF-1 plays a unique role in determining the extent of tumor invasion and recurrence^14^. Preclinical and clinical studies on HIF-1-targeting therapies have resulted in encouraging data for a number of different types of cancer^15–17^, but resultant therapies for brain tumors have so far proved elusive^14,15,18,19^.

This study presents a new HIF-1-targeting therapy specifically developed for brain tumor treatment using intratumorally delivered Acriflavine (ACF), an inexpensive, FDA-approved small molecule with HIF-1-inhibiting properties^20^. ACF is known for its trypanocidal, antibacterial, and antiseptic activity and is used topically for wound healing as well as systemically for the treatment of gonorrhea^21^. The effects of ACF on cancer cells were first reported 50 years ago^22^, but research interest peaked in 2009, when a study of 336 drugs singled out ACF as the most potent HIF-1α inhibitor with effective tumor growth inhibition in a prostate cancer model^20^. Further investigations of this small molecule resulted in highly promising antitumor activity against a wide spectrum of cancers, including colorectal^23^, perihilar cholangiocarcinomas^24^, hepatocellular^25^, and ovarian and chronic lymphocytic leukemia cancer cell lines^26^. Recently ACF has also been shown to restore drug sensitivity *in vitro* in sorafenib-resistant hepatoblastoma and gemcitabine-resistant pancreatic cell lines^27^.

Here, we present the antitumor activity and biological effects of ACF against malignant brain cancer both *in vitro* and *in vivo*. *In vitro*, ACF induced apoptosis and interfered with HIF-1α survival pathways in several brain cancer cell lines. *In vivo*, ACF was effectively delivered intracranially via biodegradable wafers and significantly improved survival resulting in nearly 100% long term survival. Our findings introduce locally delivered ACF as an effective and promising therapeutic option and show that HIF-1α-targeting molecular therapy has the potential to mark a new step forward in the treatment of brain cancer.

## Results

### Acriflavine Induces Cytotoxicity and Promotes Apoptosis in Brain Cancer Cell Lines

**Figure 1:**
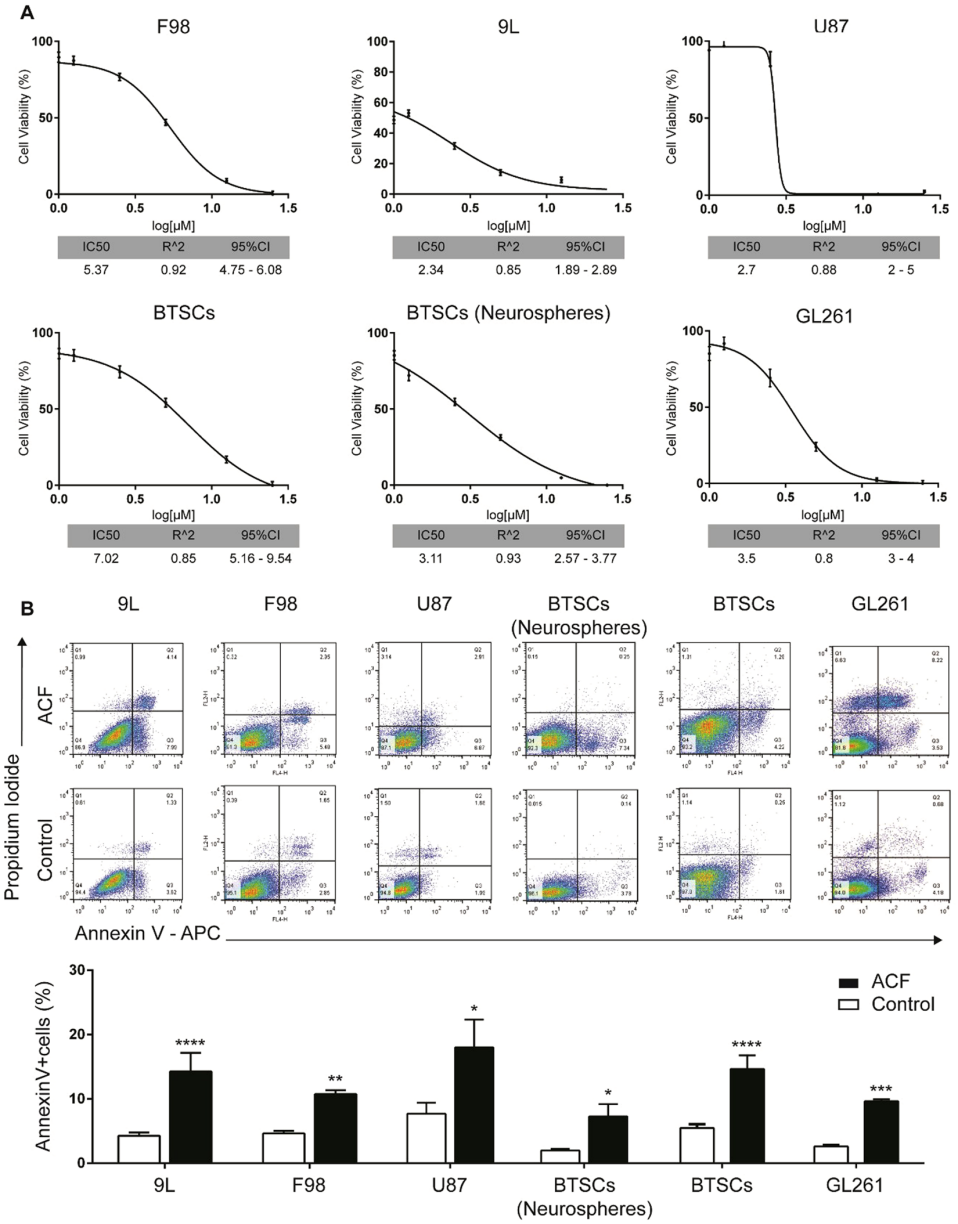
ACF increases cell death and induces apoptotic pathway in brain tumor cancer cell lines. **(A)** ACF treatment inhibits cell viability in a dose-dependent manner in 9L, F98, U87, GL261, and the two BTSC lines. Cells were incubated with increasing doses of ACF (0–25 µM) for 24 h and analyzed for cell viability. The mean inhibitory concentration 50% (IC 50) with the 95% confidence interval (95% CI) and coefficient of determination (R^2^) of the linear regression is reported for each cell line. (**B**) Activation of apoptosis in ACF-treated brain cancer cells by Annexin-V/Propidium Iodide flow cytometric analysis. Cells were stained with Annexin-VAPC-conjugated and PI, following treatment with 5Mm of ACF and analyzed by flow cytometry. Representative dot plots showing the early apoptotic cells in the lower right quadrant (Annexin-V+/PI−) and late apoptotic cells in the upper right (Annexin-V+/PI+) in different brain cancer cell lines analyzed with and without ACF treatment. Bar graphs showing that ACF significantly increases the % of Annexin-V positive cells. The data represent the means ± SE of the three different experiments (*P < 0.05, **P < 0.01, ***P < 0.001, ****P < 0.0001).

To investigate the efficacy of ACF against brain tumor cancer cells, the drug was tested in several glioma models, including historically established experimental rodent and human glioma cell lines (F98, 9L, GL261, U87) as well as human primary brain tumor stem cells (BTSCs), including both adherent cell lines and neurospheres. All cell lines were treated with increasing concentrations of ACF for 24 h; cell viability was quantified by the CCK-8 assay. ACF significantly reduced cell viability in all six brain tumor cell lines in a dose-dependent manner, with the mean inhibitory concentration 50% (IC50) ranging from 2 to 3.5 µM for 9L, GL261, U87, neurospheres,, and 5.37 and 7.02 µM for F98 and BTSCs, respectively (Fig. 1A). Similarly, ACF treatment markedly decreased cell proliferation and viability in pediatric brain tumor models. ACF was also tested on ONS-76 medulloblastoma cells and BT12 atypical teratoid/rhabdoid cells, respectively, the most common and the most aggressive malignant brain tumors in children (Fig. S1). To evaluate whether the inhibition of glioma cell proliferation was attributed to apoptosis induced by ACF, we examined apoptotic profiles in 9L, F98, U87, GL261 and the two BTSC lines using Annexin –V/Propidium Iodide flow cytometric analysis. As shown in Fig. 1B ACF treatment led to an increase of both early apoptotic and late apoptotic cells resulting in a significantly higher percentage of apoptotic cells compared to the untreated controls, nearly 2-fold for both F98 (*P* =< 0.01) and U87 (*P* =< 0.05), and 3-fold for 9L, BTSCs (*P* =< 0.0001), GL261 (*P* =< 0.001) and neurosphere BTSCs *P* =< 0.05). Notably, ACF treatment induced selective early apoptosis in the primary glioma neurosphere line in a significant manner.

### Acriflavine Reduces the Expression of Downstream Targets of HIF-1α

**Figure 2:**
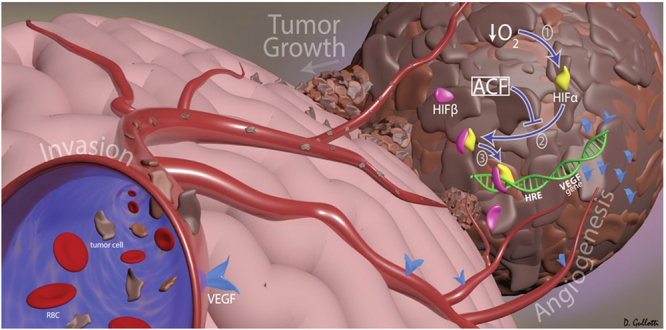
Graphical 3D-Representation of the pathways involved in glioma tumorigenesis mediated by Hypoxic-Inducible Factor. Hypoxia is a pathological hallmark of glioblastoma. The figure shows a simplified representation of the mechanisms active inside the tumor following the deficit of O_2_. Hypoxia activates HIF-1α-mediated pathways: neoangiogenesis, tumor growth and invasion which are essential to promote and maintain tumor progression and fitness. ACF inhibits HIF-1α transcriptional activity by disrupting the dimerization of HIF-1α and HIF-1β and impairing the following HIF-1 target gene expression.

**Figure 3:**
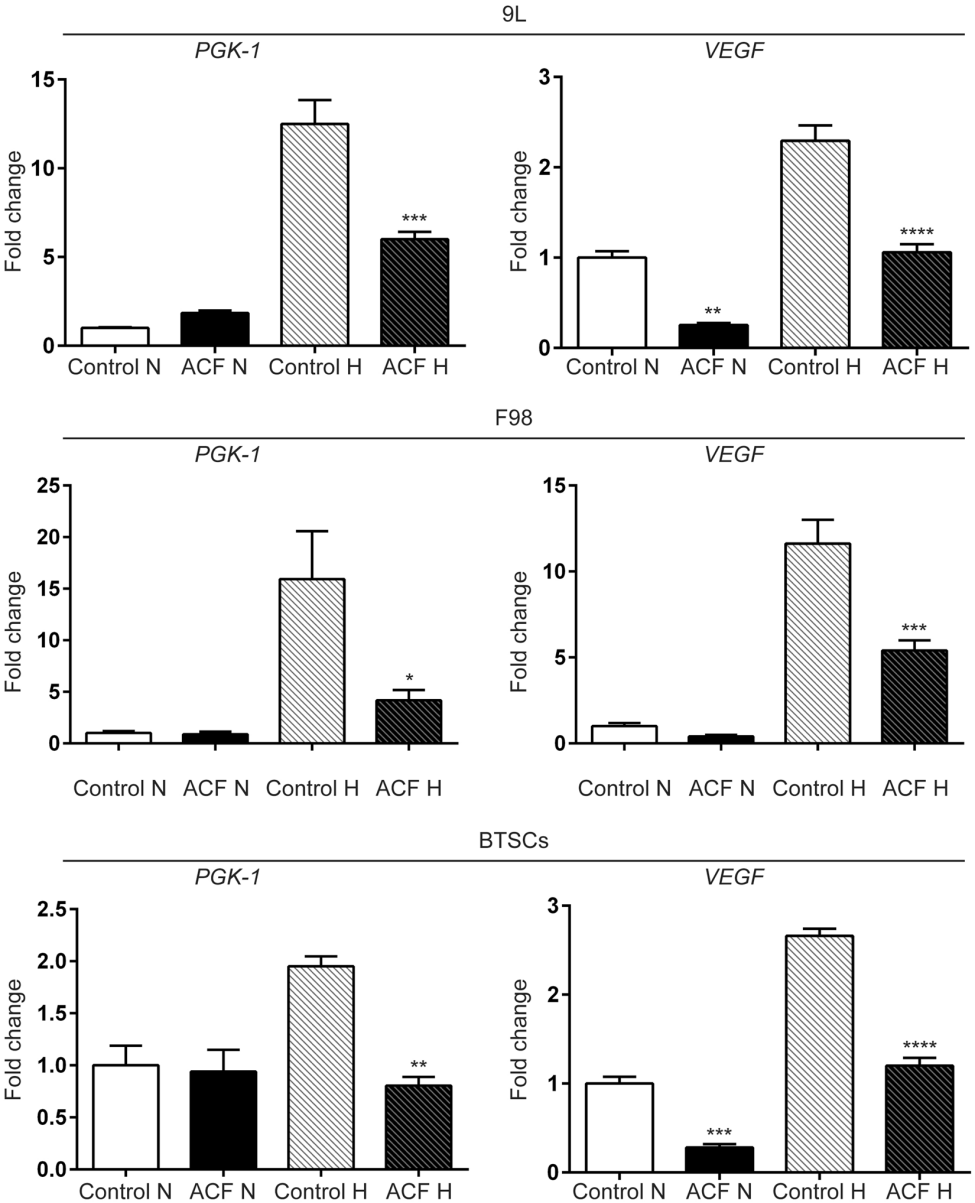
Acriflavine (ACF) decreases downstream genes of HIF-1s in brain cancer cells. Acriflavine significantly reduces PGK-1 and VEGF mRNA expression induced by hypoxia compared with untreated cells. VEGF mRNA level were reduced by ACF also under normoxia in 9L and BTSCs. Graphs representing PGK-1 and VEGF mRNA levels were assessed by qRT-PCR, in 9L, F98 and BTSCs cells with and without ACF treatment (ACF vs. control) under 20% and 1% of O_2_ (N = normoxia, H = hypoxia). The data represent the means ± SE of the three different experiments (*P < 0.05, **P < 0.01, ***P < 0.001).

**Figure 4:**
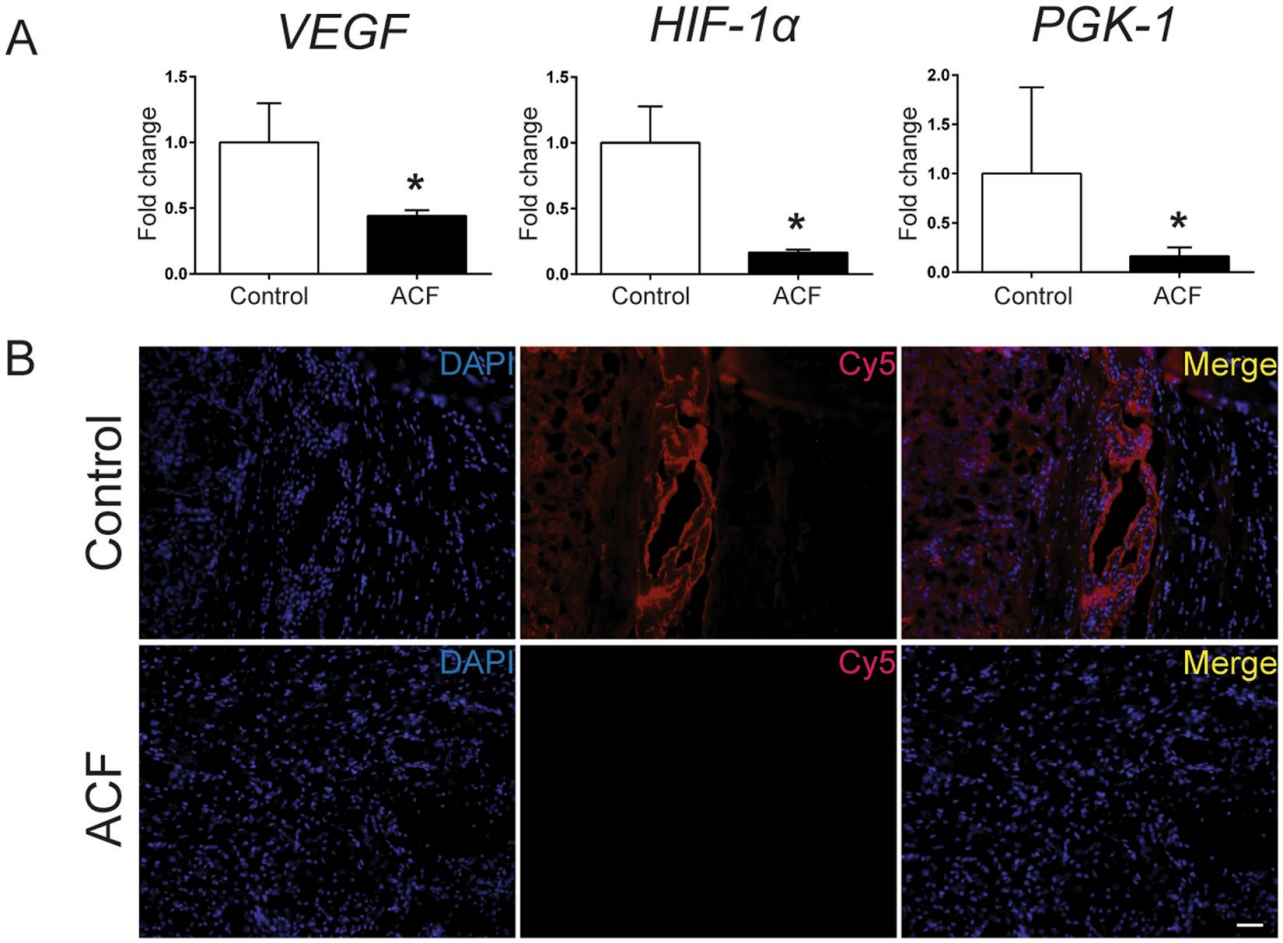
Acriflavine (ACF) downregulates the expression of HIF-1a and its downstream target genes *in vivo*. **(A)** VEGF and PGK-1 as well as HIF-1a expression were significantly reduced in the brain extracts of the rats treated with 25% ACF wafers compared to the untreated rats (control) (*P* 0.05) Means ± SE is plotted (n = 4), *P < 0.05. **(B)** Representative immunofluorescence images show no detectable VEGF expression in brain samples of animals treated with local ACF vs positive VEGF signal expressed around vessels of untreated tumor brains. At 15 days after tumor implantation, brain samples of rats treated locally with 25% ACF wafer were stained in the tumor region, on the left of corpus callosum for VEGF (red) and 4,6-diamidino-2-phenylindole (DAPI; blue). (Scale bar = 0.2 mm, Magnification, 20x).

The clinical and prognostic significance of HIF-1α expression in patients with glioma has been widely investigated^28^. Overexpression of HIF-1α together with several hypoxia-related proteins, and both up- and down-stream targets of HIF-1α, have been shown to be associated with a high grade of glioma and poor survival outcome^7^. Malignant glioma cells, similar to most cancer cells, adapt to hypoxia through the activation of hypoxia-inducible factors (HIFs), which are composed of an O_2_-regulated HIF-1α or HIF-2α subunit and a constitutively expressed HIF-1β subunit^29^ (Fig. 2). Specifically, under hypoxic conditions, HIF-1α protein becomes stable and acquires its transactivation activity. HIF-1α protein then interacts with its binding partner HIF-1β, and the resultant heterodimer, HIF-1, begins to induce the transcription of hundreds of well-characterized genes. These include pyruvate phosphoglycerate kinase 1 (PGK-1) and hexokinase II (HKII), involved in the glycolytic pathway^29–32^, and c-Met and VEGF, receptor tyrosine kinase drivers of the stem cell phenotype and neoangiogenesis in glioblastomas, respectively^11,16,33^. ACF has been shown to be a potent HIF-1α inhibitor, disrupting the dimerization of HIF-1α (or HIF-2α) with HIF-1β thereby inhibiting HIF-1 transcriptional activity in various cancer cell lines^20,24^ (Fig. 2). Here we investigated whether ACF acts similarly in malignant glioma by monitoring the effects of ACF at the main HIF-1 downstream targets: PGK-1 and VEGF in both brain cancer cell lines and brain tumor lysate. Three different glioma cell lines were exposed to 20% or 1% O_2_ in the presence and absence of ACF. Next, PGK-1 and VEGF mRNA levels were analyzed by quantitative reverse transcriptase polymerase chain reaction (qRT-PCR) in all the groups. Hypoxia-induced PGK-1 and VEGF expression were significantly decreased by half in ACF-treated 9L, F98 and BTSCs (*P* < 0.001, *P* < 0.01, *P* < 0.05) (Fig. 3). ACF did not show any significant effect in normoxic conditions on the PGK-1 mRNA level in the same cell lines, whereas VEGF mRNA levels were significantly reduced by ACF in normoxia in 9L and primary human BTSCs (*P* =< 0.01 and *P* =< 0.001, respectively). Moreover to further prove the effect of ACF on hypoxia-related proteins, VEGF, PGK-1 and HIF-1α mRNA levels were quantified in brain extracts from 9L tumor-bearing rats via RT-PCR. The brain extracts of the rats treated with 25% ACF wafers showed a significant reduction in VEGF and PGK-1 as well as HIF-1α expression compared to the untreated rats (*P* =< 0.05) (Fig. 4A). Immunostaining for VEGF confirmed the reduction of its expression in the tumor samples of animals treated with ACF (Fig. 4B).

### Release of ACF from Biodegradable Wafers

**Figure 5:**
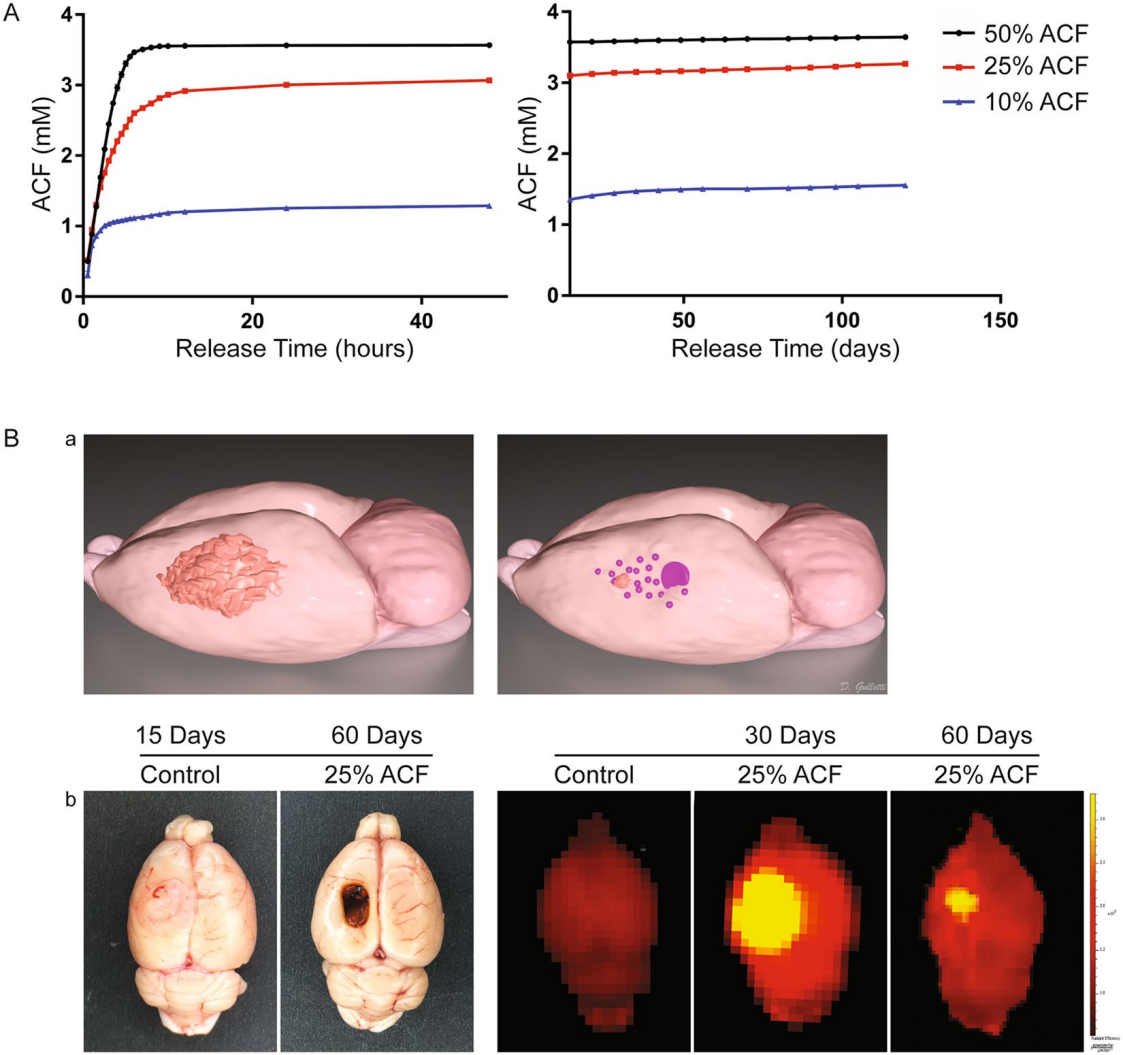
Effective release of ACF from Biodegradable Wafers *in vitro* and *in vivo*. Acriflavine is effectively released for over 100 days from the wafers (**A**) *In vitro* kinetic release profiles of 10%, 25% and 50% ACF wafers measured using the fluorescence intensity of the drug. The curves show the amount of drug released in 1 ml vials containing ACF wafers in PBS measured at close time points during the first phase of release from (0.5 to 48 hours) and weekly during the second phase up until 120 days. Mean ± SE of three separate experiments are plotted (n = 3). (**B**) *Ex vivo* imaging of the brain implanted intracranially with 25% ACF wafers confirmed the release of drug *in vivo*. Representative 3D cartoon showing the release of ACF from the intratumoral implanted wafer and the following reduction of the tumor mass (a) At 30 days the fluorescence intensity was higher compared to 60 days when the polymer was almost dissolved as and no tumor is macroscopically visible. The brains of the animals no receiving ACF (control) were fluorescent negative (b).

The blood brain barrier (BBB) is poorly permeable to hydrophilic agents. Therefore to allow an effective delivery of ACF into the tumor, CPP:SA biodegradable polymer, clinically used to deliver carmustine (bis-chloroethylnitrosourea, BCNU) was combined with ACF for local sustained delivery. ACF was combined with polymer in different ratios by weight, resulting specifically in 10% ACF, 25% ACF and 50% ACF. *In vitro* release studies were then performed to test the various profiles of drug release. As expected, due to the properties of the polymer, the majority of the drug was released within the first 24 hours (first rapid phase), the remaining drug was then released in small increments for 120 days at concentrations dependent upon the initial amount of drug used (Fig. 5A). As expected, the 50% ACF polymers released the greatest amount of compound with 3.64 mM ACF, followed by the 25% ACF polymers with 3.26 mM ACF, and the 10% ACF polymers delivering 1.56 mM ACF. *In vivo* ACF release from the wafers was confirmed using *ex vivo* fluorescence imaging of tumor bearing animals implanted with 25% ACF wafer (Fig. 5Ba). Specifically the brains of ACF-treated animals showed fluorescence intensity at 30 days and also at 60 days demonstrating that ACF is actively released and is homogeneously dispersed at the tumor site and surrounding area (Fig. 5Bb). At 60 days post implant the ACF was still detectable by fluorescence and the polymer matrix was almost entirely degraded.

### Local Therapy with ACF Wafers Improves Survival *In Vivo*

**Figure 6:**
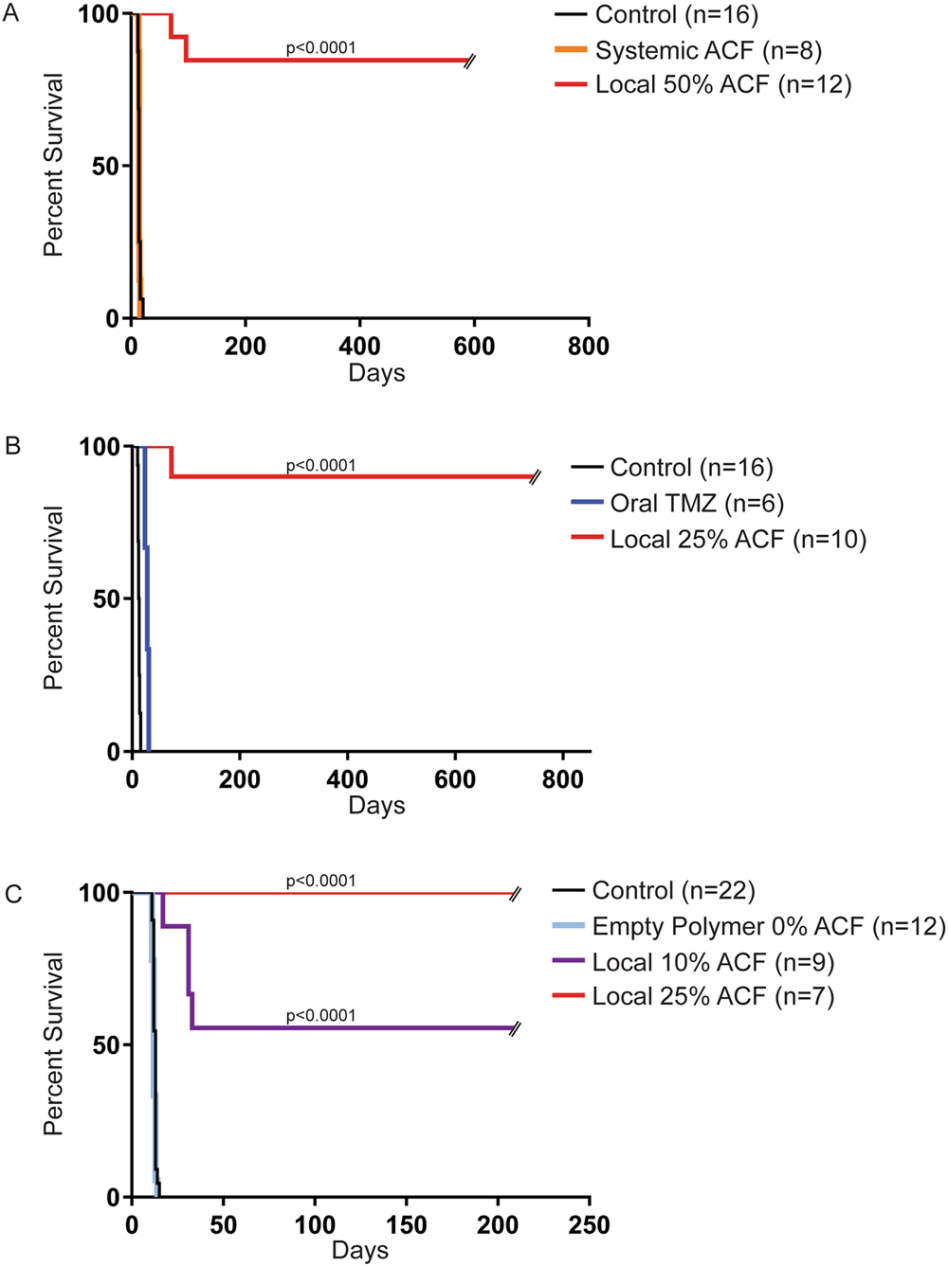
Intracranially ACF implanted wafers provide near to 100% long term survival *in vivo*. **(A)** Local 50% ACF polymers effectively improves survival compared to systemic ACF. Kaplan-Meier plots of F344 rats implanted with 9L glioasarcoma and either given no treatment (9L Control, n = 16); 5 mg/kg/day of systemic ACF (Systemic ACF Day 0, n = 8); or intracranial implantation on Day 0 of 50% ACF polymers (Local 50%ACF, n = 12). The median survival of the group receiving Local 50% ACF is significantly longer compared to the control and both groups treated with systemic ACF (*P* < 0.0001) with 83% of long term survival beyond 2 years. (**B**) Local 25% ACF polymers extend survival significantly compared to oral TMZ. Kaplan-Meier plots of F344 rats implanted with 9L gliosarcoma and either given no treatment (9L Control, n = 16); 50 mg/kg/ day of oral TMZ from day 5–9 (Oral TMZ, n = 6); intracranial implantation on Day 0 of 25% ACF polymers (Local 25%ACF, n = 10). The median survival of the group receiving Local 25% ACF is significantly longer compared to both the control and oral TMZ groups (*P* < 0.0001) with 90% of long term survivals beyond 2 years. (**C**) Local 25% and 10% ACF polymers give significant benefit in survival. Kaplan-Meier plots of F344 rats implanted with 9L glioasarcoma and either given no treatment (9L Control, n = 22); intracranial implantation of empty polymers from day 0 (Empty polymers n = 12); or  intracranial implantation on Day 0 of 10% and 25% ACF polymers (Local 10% ACF, n = 9 and Local 25%ACF, n = 7 respectively). The median survival of the group receiving Local 10% ACF is significantly longer compared to both untreated and empty polymer given animals (*P* < 0.0001) with 50% of long term survival up to 200 days. The animals treated with Local 25% ACF show 100% of long term survival up to 200 days.

We next investigated the *in vivo* efficacy of local ACF using 10%, 25% and 50% ACF:CPP:SA wafers. First, to prove the efficacy of the local delivery of ACF versus its systemic administration we compared the survival of untreated 9L gliosarcoma-bearing rats with 9L gliosarcoma-bearing rats treated either locally with intracranally implanted 50% ACF wafers or rats treated systemically with intraperitoneal injections of ACF at a dosage of 5 mg/kg/day. The systemic dose was based on data obtained from a maximum tolerated dose (MTD) study performed in non-tumor bearing F344 rats (Fig. S2). In the efficacy study, all animals received intracranial glioma. The animals treated with systemic ACF showed no difference in survival compared to untreated controls (median survival, 14 days for both groups), whereas the rats treated locally with 50% ACF wafers had a significant benefit in survival resulting in an extraordinary survival outcome of 83% of the rats surviving until the end of the study (590 days post tumor implantation, *P* < 0.0001) (Fig. 6A). We also assessed the efficacy of local 25% ACF wafers compared to oral therapy of temozolomide (TMZ), one of the current clinical standards for glioma treatment. The 9L gliosarcoma-bearing rats treated with oral TMZ showed the expected gain in median survival of 2 weeks (median survival, 28 days) as compared to the controls (median survival, 14 days; *P* < 0.0001), whereas the treatment with local 25% ACF wafers resulted in 90% long term survival, living for 2 years (*P* < 0.0001). At the 2 year time point, the study was ended (746 days post-tumor implantation) (Fig. 6B). To further validate these results we performed a third study where treatment with local 25% ACF wafers was compared to both untreated 9L-bearing rats, 9L-bearing rats implanted with empty wafers (0% ACF), and 9L-bearing rats that received a 10% ACF wafer. The rats treated with local 25% ACF wafers showed a survival rate of 100% at the end of study (209 days, *P* < 0.0001)) while the rats implanted with empty wafers showed the same median survival (12 days) as the untreated rats (12 days) confirming that the presence of the empty polymeric wafers has no effect on tumor growth. The treatment with the least amount of ACF tested, 10% ACF wafers, led to a significant benefit in median survival (*P* =< 0.0001) and resulted in 50% of the group surviving until the end of the study (209 days after tumor implantation) (Fig. 6C).

The optimal dosage in this tumor model seemed to be the 25% ACF wafer which caused only one death out of 17 rats from tumor recurrence in two separate experiments. Similar to the animals treated with 25% ACF, the rats treated with 10% ACF implants were observed to be healthy and active one day post-surgery. Notably, the animals implanted with 50% ACF implanted were observed to be slightly lethargic with diminished movements after the wafer implantation, from which, however, they spontaneously and completely recovered within the first week.

### MRI Monitoring and Histological Assessment Confirm Absence of Tumor in Animals Treated with Local ACF

**Figure 7:**
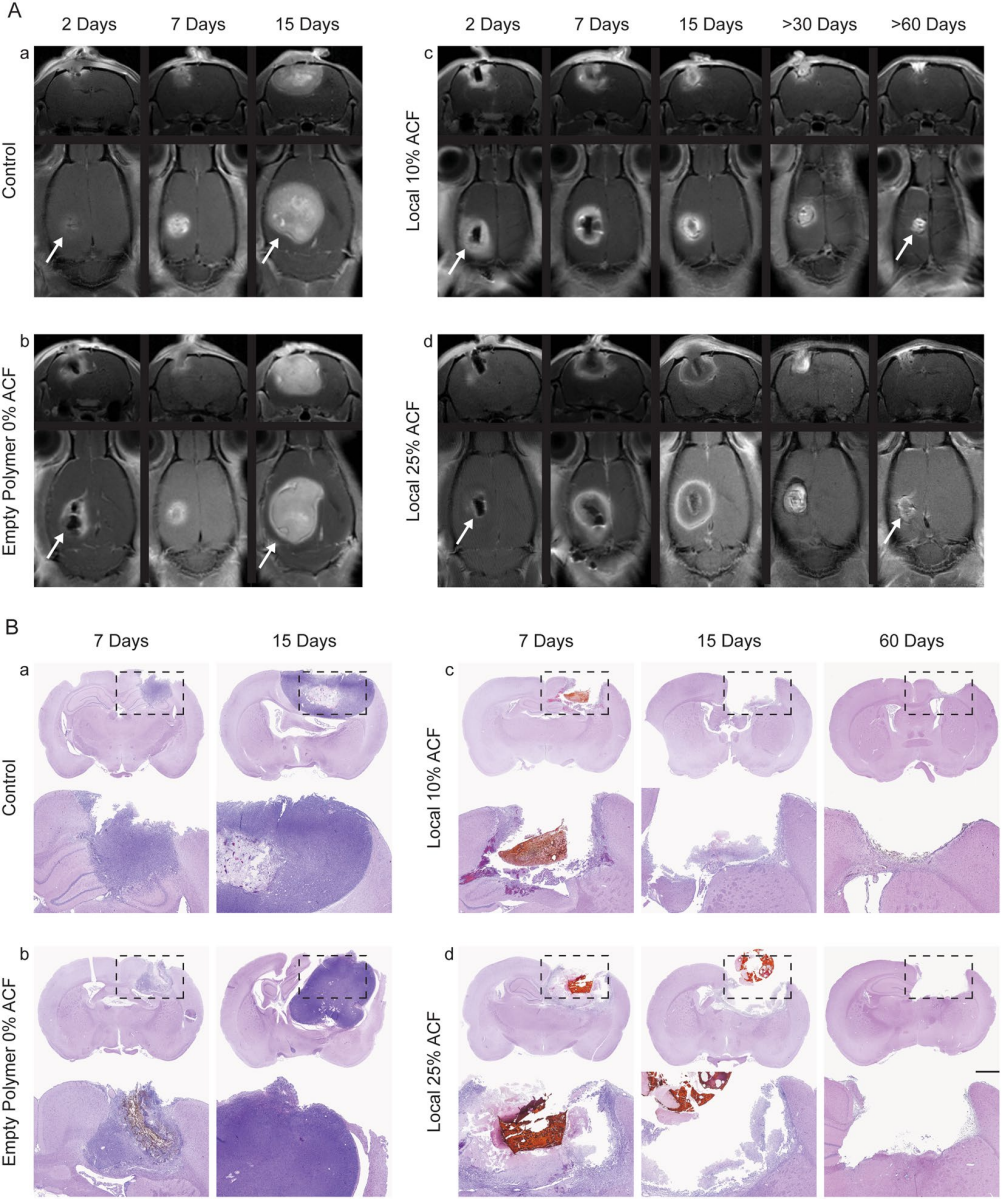
MRI follow-up scans show brain tumor regression after local treatment with ACF. (**A**) Serial axial and coronal contrast-enhanced T1-weighted images of rats implanted with gliosarcoma untreated (a) or treated with empty polymer (b) show rapid and progressive tumor growth that led them to death within 15 days. (B) The same scans performed at the survivors rats implanted with 10%ACF polymers (c) and 25% ACF polymers (d) show presence of tumor at 2 and 7 days with significant reduction of tumor mass at later follow-up scans > 30 and > 60 days, when no rats of the untreated groups were alive. (**B**) Representative coronal sections of rat brains from all the conditions (a–d) show presence of reduced tumor mass at 15 days and no tumor mass at 60 days in both groups treated with ACF wafers. Under microscopic view: presence of well- formed tumor mass at characteristic tumor cellular density in the untreated and empty polymer rats (a-b). Under microscopic view: infiltration of macrophages and presence of calcific glial reaction around the foreign body (wafer) was observed in the 10% and 15% ACF wafer treated rats together with tumor tissue at 7 and 15 days. At 60 days no viable tumor cells were detectable in either of the latter groups (c-d). (Scale bar = 1 mm, H&E Magnifications = 20x).

To validate the survival outcome, the rats in the third survival study were monitored using T1-weighted MRI over 2 months. These included animals that were untreated controls, and animals treated with 0% ACF wafers (empty polymers), 10% ACF wafers or 25% ACF wafers. Serial coronal and axial MR images pre- and post-gadolinium (Gd) administration were performed in animals at 2, 7, 15, 35, and 63 days post-implantation (Fig. 7A and Fig. S3). After intravenous administration, the T1 weighted MRI showed an aggressive and rapid growth of a homogenous tumor mass in both untreated control rats and rats implanted with empty polymer. In contrast, the post-Gd brain MRI scans showed a relatively smaller contrast enhancing tumor lesion in the ACF-treated groups compared to the untreated groups at 2, 7 and 15 days post tumor implantation. At the later time points of 35 and 63 days, the Gd-enhancement of the tumor lesion was dramatically reduced confirming the potent anti-glioma effects of ACF on the tumor mass; at these same later time points none of the untreated animals were alive for comparison (Fig. 7A). 100% and 50% of the animals implanted with the 25% and 10% ACF wafers, respectively, remained healthy and survived until the end of the study as shown in Fig. 6B,C. Since the Gd-enhancement within and surrounding the tumor lesion can also be partially attributed to neuroinflammation and necrosis, not easily distinguishable from the tumor tissue, we performed histological analysis at the same time points as the MRI (Fig. 7B). The H&E slides of these brains were examined by two blinded independent neuropathologists who confirmed the presence of tumor mass in the tumor-implanted hemisphere invading the deep brain structures at 7 and 15 days in all groups, with larger tumor masses evident in the brains of untreated rats. Glial reaction and macrophages were observed near the wafer in the 10% and 25% ACF wafer-treated rats and no viable tumor cells were detected at the 60 day time point. TUNEL assays of brain samples from rats treated with 25% ACF performed 15 days post implantation further confirmed the induction of apoptosis in the tumor mass by ACF (Fig. S4). Moreover, similarly we performed immunohistochemistry for both neuronal and astrocytes markers (anti-NeuN and anti-GFAP) showing that ACF local treatment did not affect the healthy surrounding tissue and normal brain structures (Fig. S5).

## Discussion

The HIF-1α transcriptional factor and its mediated pathways are held as the principal cause for glioma malignancy and subsequent therapeutic failure^34,35^. Already known to be crucial to glioma cell adaptation to hypoxia, HIF-1α promotes the long-term maintenance of glioma stem cells (GSCs) function - the cell population responsible for tumor self-renewal and chemoresistance^29,36^. It also boosts GSCs’ high long-term repopulation capacity by activating the hypoxic intratumoral “peri-necrotic niche” of quiescent GSCs, therefore hampering the success of anti-GCSs agents^37^. While temozolomide, the alkylating agent currently used in standard treatment for glioblastoma, has been shown to interconvert non-GCSs to GCSs thereby inducing a more aggressive phenotype and enhancing chemoresistance^38^, focusing new therapeutic approaches on impairing HIF-1α-mediated adaptation to hypoxia may be critical to disrupting the tumor microenvironment and overcoming the most aggressive features of glioma, particularly recurrence and chemoresistance^14,39,40^. In this study we show evidence of anti-glioma and other functional effects of ACF, a potent FDA-approved HIF-1α inhibitor, proving for the first time, that this small molecule provides extraordinary benefit in survival when delivered locally. ACF has already been shown, *in vitro* and *in vivo*, to inhibit the hypoxia-induced pathways underlying the polarization of tumor-associated macrophages (TAMs) toward M2 (M2-TAM), which helps to create the tumor-immunosuppressive microenvironment in gliomas. However, in this recent study the ACF treatment administrated systemically only decreased the tumor size during the first two weeks of treatment without providing any benefit in survival in glioma-implanted rodents^40^. Here, in the interest of more effective therapeutic administration, we have obtained preclinical proof of concept for the local delivery of ACF to the brain. Because ACF, a hydrophilic molecule, possesses physico-chemical and pharmacokinetic characteristics that make it difficult to cross the BBB^41^, we additionally tested the use of the biodegradable polyanhydride poly-(1,3 bis[p-carboxyphenoxy] propane-co-sebacic acid), or p[CPP:SA, 20:80]^42^, to aid in the local sustained release of ACF directly at the tumor site.

We confirmed the cytotoxic effects of ACF on a range of different malignant brain cancer cell lines, including pediatric brain cancer cells. ACF affected cell viability in a dose-dependent manner with an IC50 of 2 to 7 μM during normoxia, comparable to human leukemic monocyte lymphoma (U937) and far lower than other cancer cell lines, such as cholangiocarcinoma (SK-ChA-1), ovarian (A2780), or breast cancer (MCF-7)^24,43^. ACF treatment triggered apoptosis to a significant extent in all eight brain cancer cell lines tested, rodent and primary human BTSC, as well as pediatric brain tumor cell lines. After an analysis of the HIF-1α/HIF-1β dimerization inhibitor, we proceeded to test whether ACF affects HIF-1α and HIF-1α -related pathways in cancer cells. Our results show that ACF treatment interferes with HIF-1α -mediated pathways thereby reducing the hypoxia-induced overexpression level of PGK-1 and VEGF in glioma cell lines, both downstream targets of HIF-1α. However, VEGF levels were found to be reduced by ACF in normoxia as well. This might be explained by the fact that VEGF is also under the regulation of tumor suppressor factor p53^44^, notoriously mutant in 9L^45^ and brain tumor stem cells^46^ as well as in 28% of *de novo* glioblastomas and 65% of secondary ones^47^. Our similar analyses *in vivo* corroborated these results demonstrating a significant decrease of the expression of PGK1, VEGF, and HIF-1α after ACF treatment via RT-PCR as well as via histological analysis.

These results provide *in vitro* and *in vivo* evidence of the known anti-VEGF and anti-angiogenic effects of ACF^20^, and thus further confirm the feasibility of the future applications of this molecule against brain cancer. Several preclinical studies and clinical trials are underway to investigate the benefits of combining anti-angiogenic drugs such as (bevacizumab and sunitinib) with immune check-point blockade antibodies (such as anti-PD1, anti-PD-L1) and their preliminary results show that the combination of these promising new immunotherapeutic strategies^48–51^ can improve outcomes, especially when combined with local rather than systemic chemotherapy^52^.

Overall, our findings support the theory that ACF disrupts HIF-1α transcriptional activity and impairs the HIF-1α-mediated survival pathways of brain tumor cells. Further studies are needed to shed more light on the mechanism of ACF treatment in brain cancer cells and to determine whether its effect extends to pathways other than hypoxia-induced ones. HIF-1α is also regulated by other oncogenes, including EGFR, and loss of tumor suppression factors, such as p53 and PTEN^11^ (20–40% incidence of mutation in glioblastoma^53^); it can also be activated together with VEGF by RTK /PI3K/AKT signaling pathways frequently altered in glioblastoma (in up to 90% of cases)^54^. These results further support the recent hypothesis that HIF-1α-targeting molecular therapy may have a wide range of antitumoral biological effects^15,55^.

In this study, we assessed and confirmed that ACF can successfully inhibit tumor growth *in vivo*. Here we show that systemic administration of ACF is not effective in orthotopic glioma models, whereas intratumoral administration of ACF with biodegradable polymeric wafers provides extraordinary survival benefit and represents an effective method of ACF administration in brain cancer therapy. For the intratumoral local delivery of ACF we used p[CPP:SA, 20:80] shown to allow sustained drug release thereby improving the anti-glioma efficacy of anti-metabolic and anti-angiogenic agents^56–58^ as compared to their systemic administration. p(CPP:SA, 20:80) is an FDA-approved method of local drug delivery which is intracranially biocompatible causing no systemic or local toxicity. Currently, it is clinically utilized for the local delivery of BCNU (Gliadel®), the only intratumoral chemotherapy strategy that has been permanently added to the standard therapy for malignant gliomas^59^. Here, we show for the first time, with multiple and reproducible *in vivo* efficacy studies, the successful and significant efficacy of locally delivered ACF for the treatment of malignant gliomas. By testing intracranially implanted wafers at increasing ACF/polymer ratios, we found an optimal dosage range for ACF treatment in our preclinical model yielding extraordinary survival outcomes of 83% and 100% with rats surviving nearly 2 years after tumor implantation when treated with 50% and 25% ACF wafers, respectively. We demonstrate 50% of animals living nearly 1 year after tumor implantation when treated with 10% ACF wafers as compared to the 12 days median survival of the controls. To note, following current standard therapy, when oral temozolomide is combined with radiation therapy, according to MGMT status, the median overall survival of brain tumor patients ranges from 9 to 13.5 months^60,61^ and consistently translates into, a significant but unimpressive overall survival of 34^57^ or 36^62^ days with no long term survival, when tested in the animal model employed in this study.

*In vitro* kinetic release data demonstrate little difference in overall cumulative release between the 50% and 25% ACF polymers. The 25% ACF polymer demonstrates a slightly more graded and moderated release during the initial phase (48 hours) as compared to the precipitous release of the 50% ACF polymers. This kinetic release profile may account for the two rats treated with 50% ACF that died of tumor recurrence at 70 and 90 days after tumor implantation. The 10% ACF wafers delivered less ACF overall, and with such, their survival outcomes are significant but slightly less impressive than the 25% and 50% ACF wafers. All three loading dosages of ACF in polymer delivered millimolar quantities of ACF, a magnitude higher than the micromolar quantity necessary for the apoptosis we demonstrated *in vitro*. Additionally, in these sets of studies, we have released ACF only from the pCPP:SA polymer, therefore, a different polymer could be found to improve the ACF kinetic release profile even further.

We employed non-Gd and Gd-enhanced MRI to monitor the brain tumor growth in both treated and untreated animals as well as validate the survival data. The MR images confirmed an important radiological tumor response at the site of ACF local treatment: the tumor mass detectable in all of the rats at days 2 and 7, was very large at day 15 in the untreated rats while markedly reduced by day 30 and day 60 in the rats treated with local ACF. At the same later time points no animals were alive in the untreated groups. Of note, the residual enhancement in the 10% and 25% ACF groups may be attributed to neuroinflammation, necrosis, and residual wafer debris as the H&E performed at these time points did not show any detectable tumor tissue.

The focus of this study is to show the efficacy of an inexpensive, FDA-approved and highly promising new therapeutic for the local chemotherapy of brain cancer. The *in vivo* studies of the ACF treatment, which resulted in nearly 100% long term survival in three different *in vivo* studies, mark a unique step forward in preclinical research for brain tumor therapy. Further studies should be implemented to optimize the polymer delivery formulation to bring this safe, small molecule a step closer to clinical translation.

## Materials and Methods

### Cell lines

Human glioblastoma cell lines, U87 (ATCC HTB-14), mouse glioma cell lines, GL261(ATCC), rodent glioma cell lines, F98 (obtained from R. Barth laboratory, Ohio State University, Columbus, OH, USA), 9L gliosarcoma (obtained from the Brain Tumor Research Center, UCSF, CA, USA) were used and routinely maintained in Dulbecco’s Modified Eagle Medium (Lonza, Portsmouth, NH, USA) supplemented with 10% fetal calf serum (Lonza) at 37 C° in 5% CO_2_-humidified incubators and were subcultured once or twice a week. Human primary brain tumor stem cell neurosphere lines, GB1A(0913) and primary brain tumor stem cell lines (BTSCs) JHH 1113, respectively derived by Vescovi and colleagues or generated within the department of Neurosurgery of the Johns Hopkins University (JHU, Baltimore, MD, USA)^63,64^ within compliance of JHU regulations, were grown in NeuroCult NS-A basal medium containing NeuroCult NS-A proliferation supplements (Stem Cell Technologies), 20ng/mL epidermal growth factor (PeproTech, Rocky Hill, NJ, USA), 10ng/mL basic fibroblast growth factor (PeproTech), and 4 μg/mL heparin (StemCell Technologies, Vancouver, Canada).

### Cell Viability assay

The cells were seeded in 96-well plates at a density of 4 × 10^3^ cells/well and incubated for 24 h. The cells were cultured in an incubator with various concentrations of ACF ranging from 0 to 25 µM. After 24 h, cell Counting Kit-8 (CCK-8, Dojindo) was used to determine cell viability according to manufacturer’s protocol.

### Apoptosis Assay

Apoptotic cells were detected using an Apoptosis kit (APC-AnnexinV/Dead Cell Apoptosis Kit, (Invitrogen, Life Technologies). The cells were plated in 6-well plates at the density of 0.5 × 10^6^ cells/well and incubated for 24 h. Then, ACF was added to the cells (5 µM)^20^ and incubated for an additional 24 h. The negative control was prepared in medium with no additives. Briefly the cells were then incubated for 15–20 min in the dark at RT in 100 μL of Annexin-binding buffer 5X (10mMHEPES pH7.4, 140 mM NaCl, 2.5 mM CaCl2), containing 5 μL of Annexin V-APC and 1 μL of Propidium Iodide (PI) solution at 100 μg/mL, and then analyzed by FACS (FACS Calibur, Becton-Dickinson, Franklin Lakes, NJ, USA) within 1 h. Subsequent analyses were performed using FlowJo software (FlowJo LLC, Ashland, OR, USA).

### QRT-PCR Analysis

9L, F98 and BTSCs cells were seeded at the concentration of 5 × 10^6^ cells in T75 cm^2^ flasks and incubated at 20% and 1% O_2._ After 24 h the media was replaced with either fresh media or media containing 1 µM of ACF for 24 h. Then GPGK-1 and VEGF mRNA levels were quantified via RT-PCR. Similarly, HIF-1α, PGK-1 and VEGF mRNA levels were also measured in brain extracts. The brain lysates were obtained from 8 rats bearing 9L intracranial gliosarcoma tumor and randomized in 2 groups: 4 were treated with local 25% ACF wafers and 4 did not received treatment. Twelve days after tumor implantation, when the control rats started to show signs of sickness, both groups were sacrificed and perfused. The brains were harvested and processed separately. Total mRNAs were extracted from cells or tumor tissue with the RNeasy Mini kit (Qiagen) and reverse-transcribed using the SuperScript® III First-Strand Synthesis System (Invitrogen, Life Technologies) according to the manufacturer’s instructions. The cDNAs were amplified with iQ™ SYBR® Green Supermix on a Bio-Rad C1000 Bio-Rad thermocycler. Data were analyzed using Bio-Rad CFX Manager and normalized to untreated cells using the Cq method. Each sample was used to generate RNA at the end of the treatment and used for 3 independent qPCR reactions. Relative transcript expression levels were measured using primers (Supplementary Table 1).

### Preparation of ACF Biodegradable Polymer Wafers

Poly-[1,3 bis(p-carboxyphenoxy) propane-co-sebacic acid] p(CPP:SA, 20:80) (Eisai Inc., Woodcliff Lake, NJ) was synthesized by melt polycondensation, as previously described^65^. ACF was incorporated into the polymer matrix. Briefly, the appropriate amount of ACF to obtain the percentage of 10%, 25% and 50% of ACF:pCPP:SA by weight was dissolved with the polymer into a solution of methylene chloride. Resulting solutions were then placed in a vacuum desiccator until dried. The polymer mixtures were pressed into cylinders of 10 mg, and then stored at −20 °C.

### *In Vitro* and *In Vivo* Release of ACF from Biodegradable Polymer Wafers

10%, 25% and 50% ACF wafers were placed in separate vials (n = 6) containing 1 mL of phosphate-buffered saline (PBS) at 37 °C. Equal volumes of PBS were removed and replaced at set time-points, specifically every 0.5 hours for the first 6 hours, every hour for the first 12 hours and then every 12 hours. After the first 48 hours the time points were weekly for 12 weeks. Fluorescent intensity was then analyzed using a microplate reader (Perkin Elmer Victor 3 Wallac) following the innate fluorescence of ACF (λ_ex_ = 453 nm and λ_em_ = 507 nm). A standard curve was constructed of known concentrations of ACF and used to calculate cumulative ACF released. *In vivo* ACF release was then also confirmed using *ex vivo* fluorescence imaging of brains implanted with 25% ACF wafers. Specifically, 9L tumor bearing animals were implanted with 25% ACF wafers and brain fluorescence was imaged at 30 and 60 days post implantation. The animals were euthanized and perfused with 4% paraformaldehyde, the brains were then extracted for imaging with IVIS (Caliper Life Sciences, formerly Xenogen). Images were recorded with an exposure time of 1 minute. Signal was recorded as radiant efficiency unit using Xenogen IVIS 200 system. Tumor bearing animals euthanized at 15 days post-implantation were used as negative controls for fluorescence.

### Animal Studies

All animals were housed in standard facilities and given free access to food and water. Animal studies were approved by and conducted in accordance with the policies and guidelines of the Johns Hopkins University Animal Care and Use Committee (ACUC). For the *in vivo* studies an orthotopic 9L gliosarcoma model was used. The tumor was implanted using 9L tumor pieces, specifically, for intracranial implantation, 164 F344 rats (36 rats for the first survival study, 32 rats for the second, 50 rats for the third one, and 46 rats for the imaging and histology studies) were anesthetized and prepped as previously described. Briefly, rats were anesthetized with an intraperitoneal (IP) injection of 3 mL/kg of a stock solution containing ketamine hydrochloride, 75 mg/mL (Ketathesia, Butler Animal Health Supply; Dublin, OH); xylazine 7.5 mg/mL (Lloyd Laboratories; Shenandoah, Iowa), and 14.25% ethyl alcohol in 0.9% NaCl. The head was shaved with clippers and prepared with Prepodyne solution (West Penetone, Montreal, Canada). After a midline scalp incision, the galea overlying the left cranium was swept laterally. A 3 mm burr-hole was placed in the left parietal bone with its center 3mm lateral and 5mm posterior to bregma. Then, under surgical microscope magnification, an opening through the dura and cortex was made and a small area of cortex was resected and 2 mm^3^ piece of the tumor allograft was placed in the resection cavity. The skin was closed with surgical staples. In the polymer treatment groups, one 10%, 25% or 50% ACF wafer per animal was placed at the time of tumor implantation. The animals were allowed to awaken, then released to their cages and received lab chow and water ad libitum. All the rats were evaluated daily post-operatively and closely monitored for signs of toxicity, including failure to thrive and neurologic deficits. Rats receiving the systemic ACF treatment were treated with 5 mg/kg/day of ACF administrated intraperitoneally. Rats receiving oral temozolomide (TMZ) were dosed 50 mg/kg via daily gavage on days 5–9 following tumor implantation^57^. Survival was assessed and autopsies performed at the end of the studies. The brains were removed and immediately processed or preserved in 10% formalin according to the protocol of the following analysis.

### Histological Analysis

The brains were harvested after perfusion of the animals and fixed in freshly prepared 4% paraformaldehyde or 10% formalin for 1 day. The samples were paraffin-embedded and H&E stained by the Johns Hopkins University Histopathology Core. For assessing VEGF expression of the brain tumors after ACF treatment, the brains were fixed in 4% paraformaldehyde at 4 °C for at least 24 hr, cryoprotected by sinking in 15% and 30% sucrose in 0.1 M PBS, and then embedded in Optimal Cutting Temperature compound (OCT). Cryosection slides were prepared at 10 μm using a Leica CM1905 cryostat for immunofluorescence staining. The sections were permeabilized using PBS-Triton (0.2%), blocking was carried out using 5% blocking buffer: 10% goat serum in PBS-T. Primary antibodies were incubated in diluted (1%) blocking buffer overnight at a dilution of 1/200 at 4 °C, washed with PBS-T and incubated with secondary antibodies diluted at 1/500 in 1% diluted buffer for 1 h at room temperature, subsequently washed with PBS-T, counterstained with 4,6-diamidino-2-phenylindole 1 μg ml^−1^ in PBS for 10 min, washed with PBS, and mounted using mounting media (Vector Biolabs). Staining was done using the following antibodies and reagents: Anti-VEGF primary antibody (A-20, Santa Cruz) and Alexa-594 anti-rabbit (Invitrogen) secondary antibody.

### Magnetic Resonance Imaging Experiments

The same three rats per group from the third survival study were monitored with anatomical MR imaging (T_2_- and T_1_-weighted) at 2, 7, 15, 32 and 65 days after surgery. The animals were anesthetized using isoflurane during the imaging procedure. MRI scans were performed using a horizontal bore 4.7 T Biospec animal imager (Bruker Biospin) with an actively decoupled cross-coil setup (a 70-mm body coil for radiofrequency transmission and a 25-mm surface coil for signal reception). Specifically, high-resolution T_2_-weighted imaging with fast spin echo acquisition (echo train length, 8; repetition time, 3 s; echo time, 64 ms; 5 slices; slice thickness, 1.5 mm; number of averages, 2) was acquired in both the horizontal plane (matrix, 256 × 192; field of view, 42 × 32 mm^2^) and the coronal plane (matrix, 192 × 192; field of view, 32 × 32 mm^2^). Then, T_1_-weighted images (repetition time, 700 ms; echo time, 10 ms; number of averages, 10) with and without Gd, administered intravenously via tail injection, were acquired with the same geometry and location as the T_2_-weighted images.

### Statistics analysis

All statistical analyses were carried out using GraphPad Prism Software (Version 6.0, GrapPad Software, San Diego, CA). One-way ANOVA with Bonferroni or Tukey post-tests or a non-parametric Kruskal-Wallis test were performed, depending on the type of distribution of the data. Data distribution was determined using the D’agostino test; graphs represent the mean ± SEM. For survival studies, survival was analyzed using the Kaplan-Meier estimator, and statistical significance was established using log-rank analysis.

### Data availability

All cell lines and materials included in these studies will be freely shared with any interested party. The data that support the findings of this study are available from the authors.

## Electronic supplementary material

Supplementary Info
