# Supplementary material for: HIF-1α- Targeting Acriflavine Provides Long Term Survival and Radiological Tumor Response in Brain Cancer Therapy
Source: Sci Rep. 2017 Nov 2;7:14978. doi: 10.1038/s41598-017-14990-w (PMC5668269; doi:10.1038/s41598-017-14990-w)
Supplement: Supplementary file 1 — Supplementary Info [file 41598_2017_14990_MOESM1_ESM.pdf]

HIF-1α- Targeting Acriflavine Provides Long Term Survival and Radiological Tumor Response in Brain Cancer Therapy

Antonella Mangraviti[1], Tula Raghavan[1], Francesco Volpin[1], Nicolas Skuli[1], David Gullotti[1], Jinyuan Zhou[2], Laura Asnaghi[3], Eric Sankey[1], Ann Liu[1], Yuan Wang[1], Dong-Hoon Lee[2,9], Noah Gorelick[1], Riccardo Serra[1], Michael Peters[1], Destiny Schriefer[1], Fabien Delaspre[4], Fausto J. Rodriguez[3], Charles G. Eberhart[3,5,6], Henry Brem[1,5,6,7], Alessandro Olivi[1,5,8], and Betty Tyler*[1]

[1]Department of Neurosurgery, Johns Hopkins University, Baltimore, MD; [2]Department of Radiology, Johns Hopkins School of Medicine, Baltimore, MD; [3]Department of Pathology, Johns Hopkins School of Medicine, Baltimore, MD; [4]McKusick-Nathans Institute for Genetic Medicine, Johns Hopkins University, Baltimore, MD; [5]Department of Oncology, Johns Hopkins University, Baltimore, MD; [6]Department of Ophthalmology, Johns Hopkins University, Baltimore, MD; [7]Department of Biomedical Engineering, Johns Hopkins University, Baltimore, MD; [8]Department of Neurosurgery, Catholic University School of Medicine, Rome, Italy; [9]Faculty of Health Sciences and Brain & Mind Centre, The University of Sydney, Sydney, NSW, Australia

***Corresponding Author**

Betty Tyler (btyler@jhmi.edu)

SUPPLEMENTARY FIGURES

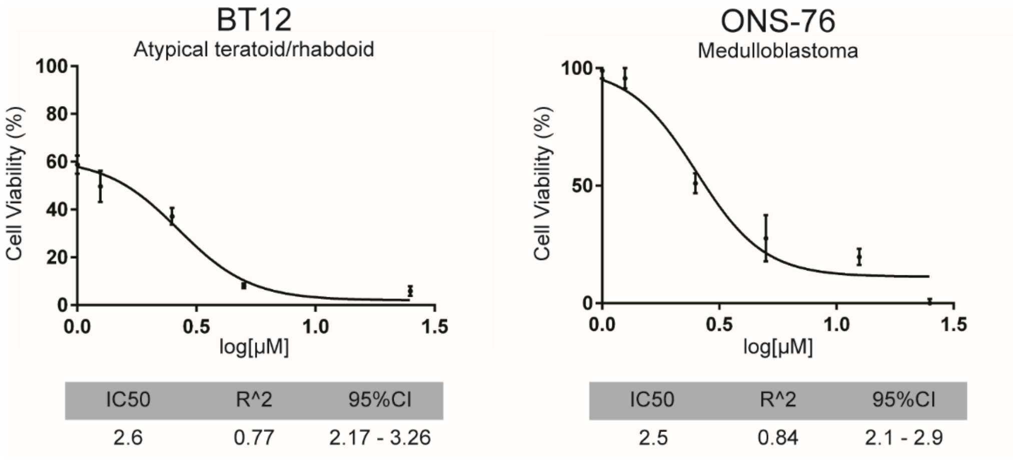

Supplementary Figure 1. Acriflavine induces cell death in pediatric brain cancer cells
BT12 and ONS-76 cells were incubated with increasing doses of ACF (0-25μM) for 24h and analyzed for cell viability. The mean inhibitory concentration 50% (IC 50) with the 95% confidence interval (95% CI) and coefficient of determination (R^2) of the linear regression is reported for each cell line. Three experiments with at least 5 replicates were performed for each cell line.

Supplementary Table 1. Quantitative-PCR primer sets

Target Genes	Species	Sense	Anti-Sense
Gapdh	Rat	5′-ACTCCCATTCCTCCACCTTT-3′	5′-TTACTCCTTGGA-GGCCATGT-3′
β-Actin	Rat	5'-AAGTCCCTCACCCTCCCAAAAG-3'	5'-AAGCAATGCTGTCACCTTCCC-3'
	Human	5′-CCCAGCACAATGAAGATCAA-3′	5′-GATCCACACGGAGTACTTG- 3′
Hprt	Rat	5'-CTCATGGACTGATTATGGACAGGAC-3'	5'-GCAGGTCAGCAAAGAACTTATAGCC-3'
	Human	5'- TGAGGATTTGGAAAGGGTGT-3'	5'-GAGCACACAGAGGGCTACA-3'
Vegf	Rat	5′-TGCCAAGTGGTCCCAG-3′	5′-CGCACACCGCATTAGG-3′
	Human	5'- AAATGCTTTCTCCGCTCTGA -3'	5'- CCCACTGAGGAGTCCAACAT -3'
Pgk-1	Rat	5'-ATGCAAAGACTGGCCAAGCTAC-3'	5'-AGCCACAGCCTCAGCATATTTC-3'
	Human	5′-AGTACATATGTCGCTTTCTAACAAGCTG-3′	5′-AGTAGGATCCCTAATGCCAAGTGGAGATGCA-3′

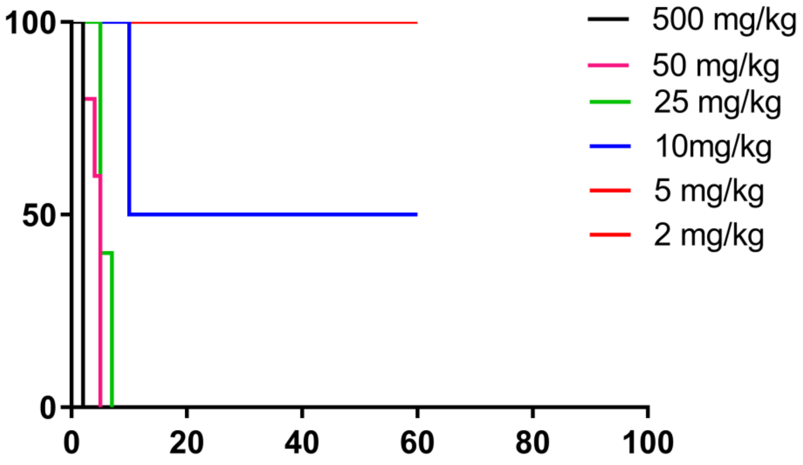

Supplementary Figure 2. MTD study for systemic administration of ACF in F344 rats
The systemic administration of 5 mg/kg/day given intraperitoneally was found to be the highest dosage of ACF administrated safely in F344 rats. The Kaplan-Meier plot shows six increasing dosages of ACF tested (3 rats per group). The groups treated with 2 or 5 mg/kg/day had no deaths, therefore, the highest dosage of 5 mg/kg/day was considered the MTD of systemically administered ACF.

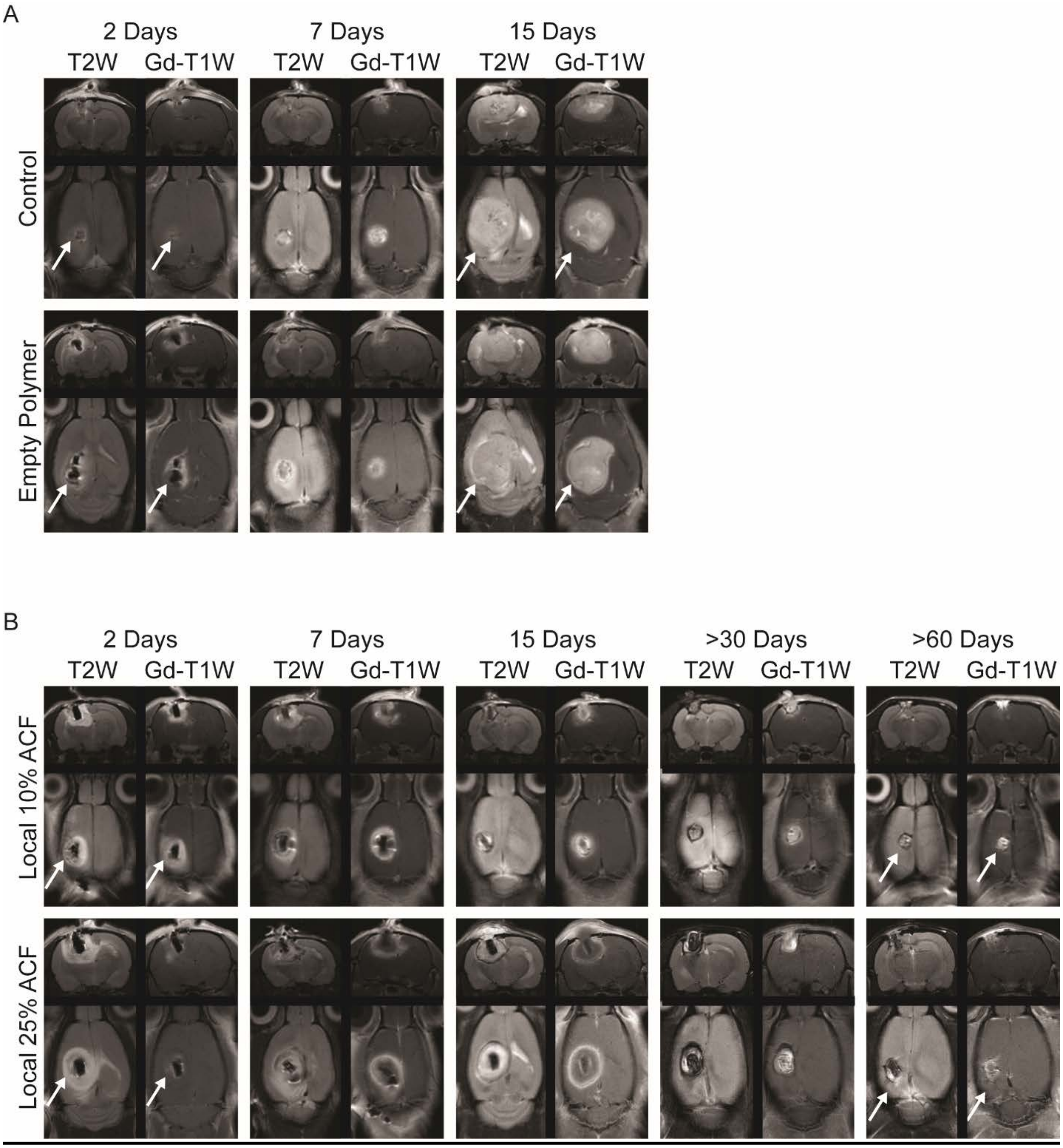

Supplementary Figure 3. Representative T$_2$-weighted (T2W) and gadolinium-enhanced T$_1$-weighted (Gd-T1W) MRI scans, both coronal (top) and axial (bottom), demonstrate the progressive brain tumor response to the local treatment of ACF.

(**A**) The MRI scans of the untreated rats (control and empty polymer 0% ACF) show a continuous increase in the size of Gd-enhancing tumor mass up until 15 days. At the later time points none of the untreated rat were alive for MRI comparison. (**B**) The serial imaging of ACF-treated rats (10% ACF and 25% ACF) shows a progressive decrease of the Gd-enhancing area at

30 and 60 days. On serial T2W and Gd-T1W images, the hyperintensities (white arrows) in the tumor cavity suggest the presence of tumor mass and its progressive growth/reduction, as well as reactive neuroinflammation due also to the foreign body reaction especially at the early time-points after the surgery.

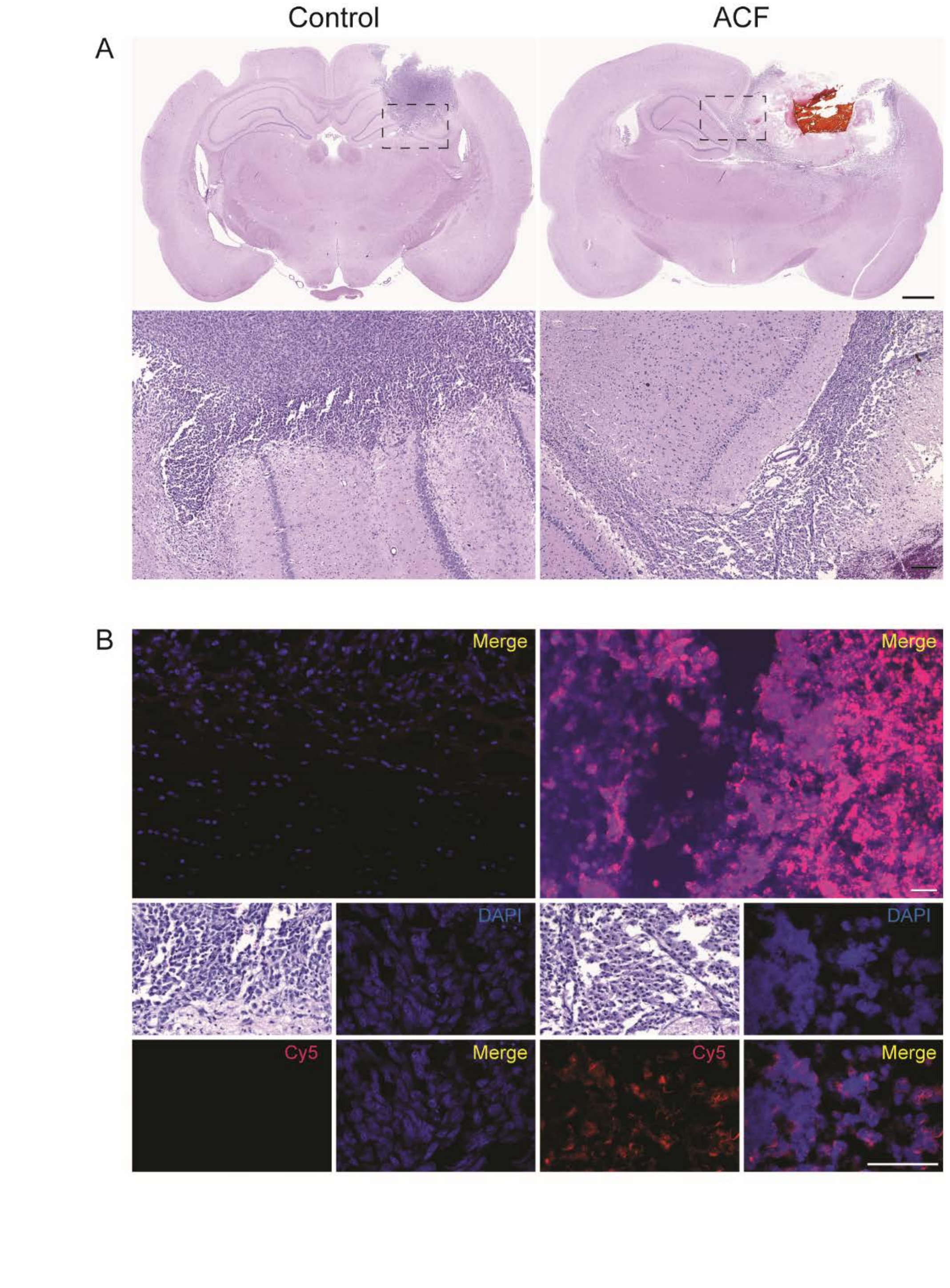

Supplementary Figure 4. ACF wafers induce apoptosis in brain tumor *in vivo*.

(A) Representative H&E coronal sections of the brains of the untreated and 25% ACF – implanted rats (Scale bar=1 mm, Magnification 20x scale bar = 200 µm). (**B**) Representative immunofluorescence images show apoptotic TUNEL-positive cells in the tumor area of the ACF-treated animals vs. TUNEL-negative cells in the control. TUNEL staining (red) in brain tumor samples of 9L bearing rats untreated and treated with 25% ACF wafer at 15 days. Nuclei were stained with DAPI (blue). (Scale bar=0.2 mm, Magnifications= 20x and 63x).

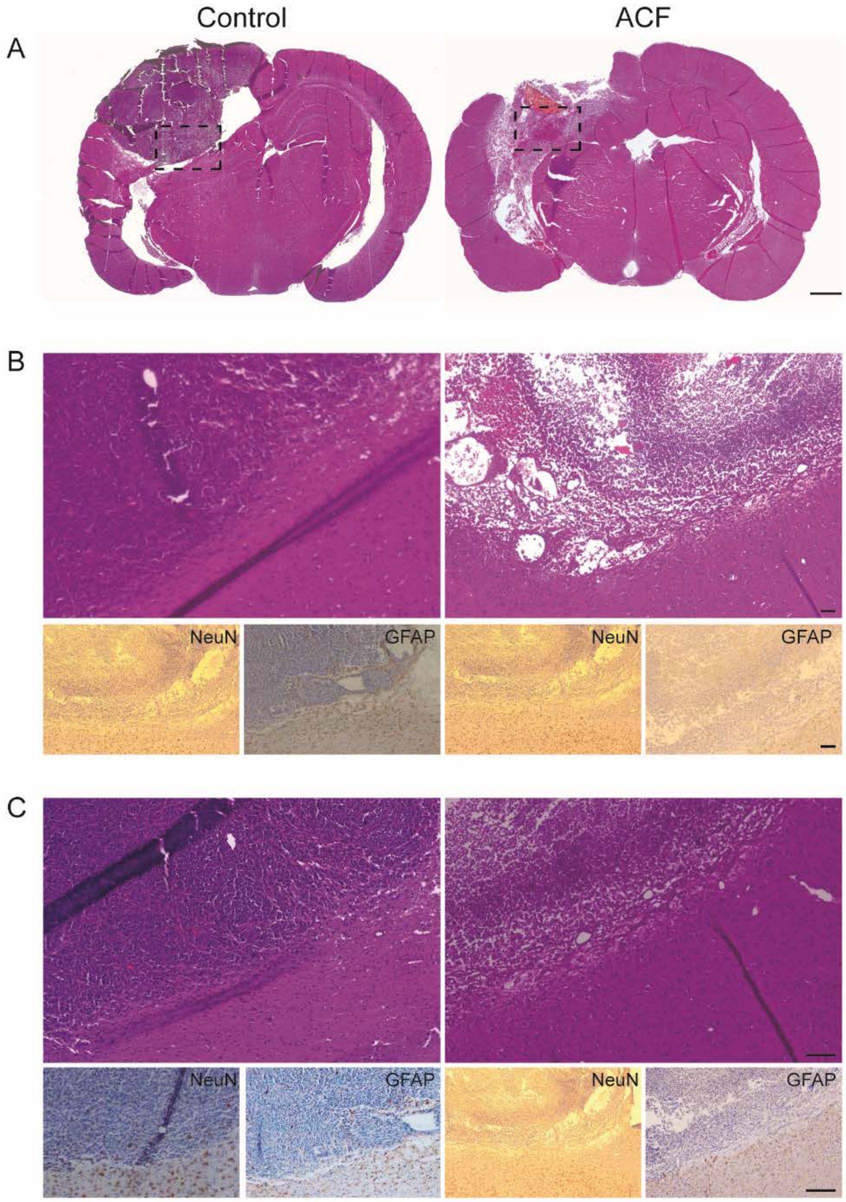

Supplementary Figure 5. ACF wafers have no neurotoxic effects in the tumor surroundings *in vivo*.
(**A**) Representative H&E coronal sections of the brains of the untreated and 25% ACF - implanted rats (Scale bar=1mm). **(B) (C)** Representative images of the immunohistochemistry of the tumor surrounding area of ACF-treated and untreated rats stained for neuronal (NeuN) and astrocyte (GFAP) markers showing no evidence of neurotoxic effects of the ACF-wafers on the normal brain cells (B: Magnification 20x, C: Magnification 40x, scale bar 0.2 mm)

SUPPLEMENTARY INFORMATION

Experimental animals

Female F344 rats, each weighing 160 to 200g, were purchased from Harlan Bioproducts (Indianapolis, IN) and housed by the Hunterian Neurosurgical Laboratory in standard facilities at the Johns Hopkins University and provided free access to rodent chow and Baltimore City water. To establish and maintain the orthotopic gliosarcoma model using 9L tumor pieces, 9L gliosarcoma tumor xenograft was maintained as a subcutaneous mass passaged every 3-4 weeks in the flanks of F344 rats. For intracranial implantation then, the tumor was surgically excised from the carrier animal and sliced into $2mm^3$ allografts for their intracranial tumor implantation. All animals were treated in accordance with the policies and guidelines and the experiments performed were approved by the Institutional Animal Care and Use Committee at the Johns Hopkins University.

Determination of the maximum tolerated dosage (MTD) of systemic Acriflavine

Eighteen healthy F344 rats, three per dosage, received daily intraperitoneal administration of Acriflavine at the following dosages: 2, 5, 10, 25, 50, 500 mg/kg. Animals were observed daily for a maximum time of 120 days, and survival was recorded. The animals included in the groups with the highest dosages (25, 50, 500 mg/kg/day) died or needed to be euthanized due to signs of

systemic toxicity. One out of 3 rats died in the group of 10 mg/kg/day, and no rats died in the 2 and 5 mg/kg/day groups. 5 mg/kg/day systemic ACF resulted in the MTD dosage in our model and therefore was safely used for the subsequent efficacy experiments.

Tunel Assay

The brains were collected and fixed in freshly prepared 4% paraformaldehyde or 10% formalin for 1 day. For the TUNEL assay, used to detect apoptotic tumor cells after ACF treatment, the slides were deparaffinized in xylene and rehydrated through a gradient alcohol series. The TUNEL staining was then performed in accordance with the manufacturer's protocol (Click-iT® Plus TUNEL assay Alexa 594, Life Technologies). Nuclei of tumor cells were stained with 4', 6-Diamidino-2-Phenylindole, Dihydrochloride, DAPI (ThermoFisher).

Immunohistochemistry

Immunohistochemical staining was performed on 10-μm-thick sections as previously described[1] Briefly, the slides were deparaffinized and antigen retrieval was performed by incubating the slides in hot sodium citrate, pH 6.0, for 30 min. Slides were incubated for 10 min in 3% hydrogen peroxide, washed in distilled H_2O, and incubated for 30 min with 0.4% Triton X100/TBS pH 7.2. Non-specific binding was blocked by treating the slides with 4% normal goat serum in 0.1% Triton X100/TBS for 1 h. Slides were then incubated overnight in the following rabbit primary antibodies:Anti- NeuN (Neuronal Marker, Abcam, #ab104225, 1:500), Anti-GFAP(Glial Fibrillary Acidic Protein, Dako#Z0334, 1:500). Secondary antibodies were purchased from Vector Laboratories, Burlingame, CA, USA (anti-rabbit: #PK-6101; anti-mouse: #PK6102) and diluted 1:200 in 2% normal goat serum/0.1% Triton X100/TBS.

Diaminobenzidine was used as chromogen and slides were counterstained with hematoxylin before mounting.

References

1. Mahale A, *et al.* Altered gene expression in conjunctival squamous cell carcinoma. *Modern pathology : an official journal of the United States and Canadian Academy of Pathology, Inc* 29, 452-460 (2016).
